# Case Report: Fetoscopic Laparoschisis (FETO-LAP)—A New Therapeutic Route to Explore for Fetuses with Severe Diaphragmatic Hernias

**DOI:** 10.3390/children10111758

**Published:** 2023-10-30

**Authors:** Thomas Kohl, Nadja Riehle, Leila Messroghli, Sibylle Maus, Christiane Otto, Michaela Klinke, Richard Martel, Grietje Beck, Michael Boettcher, Thomas Schaible

**Affiliations:** 1 Deutsches Zentrum für Fetalchirurgie & Minimal-Invasive Therapie (DZFT), Theodor-Kutzer-Ufer 1-3, 68167 Mannheim, Germanysibylle.maus@gmx.net (S.M.); 2 Department of Anesthesiology, Theodor-Kutzer-Ufer 1-3, 68167 Mannheim, Germany; leila.messroghli@umm.de (L.M.); grietje.beck@umm.de (G.B.); 3 Department of Obstetrics & Gynecology, Theodor-Kutzer-Ufer 1-3, 68167 Mannheim, Germany; christiane.otto@umm.de; 4 Department of Pediatric Surgery, Theodor-Kutzer-Ufer 1-3, 68167 Mannheim, Germany; michaela.klinkepetrowsky@umm.de (M.K.); richard.martel@umm.de (R.M.); michael.boettcher@medma.uni-heidelberg.de (M.B.); 5 Department of Neonatology, Mannheim University Hospital (UMM), Theodor-Kutzer-Ufer 1-3, 68167 Mannheim, Germany; thomas.schaible@umm.de

**Keywords:** fetal surgery, fetal intervention, fetoscopy, diaphragmatic hernia, FETO, tracheal balloon occlusion, laparoschisis, experimental surgery, new techniques

## Abstract

Background: The purpose of this report is to describe the seminal case of a near-term human fetus with a life-threatening left diaphragmatic hernia that underwent fetoscopic tracheal occlusion (FETO) combined with fetoscopic partial removal of herniated bowel from the fetal chest by fetoscopic laparoschisis (FETO-LAP). Case summary: A life-threatening left diaphragmatic hernia (liver-up; o/e LHR of ≤25%; MRI lung volume ≤ 20%) was observed in a human fetus at 34 weeks of gestation. After counselling the mother about the high risks of postnatal demise if left untreated, the expected limitations of fetoscopic tracheal occlusion (FETO), and the previously untested option of combining FETO with fetoscopic laparoschisis, i.e., partial removal of the herniated bowel from the fetal chest (FETO-LAP), she consented to the latter novel treatment approach. FETO-LAP was performed at 36 + 5 weeks of gestation under general maternofetal anesthesia. Mother and fetus tolerated the procedure well. The neonate was delivered and the balloon removed on placental support at 37 + 2 weeks of gestation. On ECMO, a rapid increase in tidal volume was seen over the next eight days. Unfortunately, after this period, blood clots obstructed the ECMO circuit and the neonate passed away. Discussion: This seminal case shows that in a fetus with severe left diaphragmatic hernia, partial removal of the herniated organs from the fetal chest is not only possible by minimally invasive fetoscopic techniques but also well tolerated. As the effect of FETO alone is limited in saving severely affected fetuses, combining FETO with fetoscopic laparoschisis (FETO-LAP) offers a new therapeutic route with multiple, potentially life-saving implications.

## 1. Introduction

Fetoscopic tracheal balloon occlusion (FETO) has proven its efficacy in randomized trials to improve survival in fetuses with severe diaphragmatic hernia [1,2]. However, FETO has limited effects, and, more than two decades after its clinical introduction in Europe, the survival rates of severe cases still linger between 40% and 70%. Therefore, finding new routes and strategies for their prenatal treatment is important.

In our experience, particularly fetuses with left diaphragmatic hernia, presenting with large volumes of herniated liver and bowel within their chest, accompanied by a far posteriorly displaced and distended stomach and preferential streaming of ductus venosus and inferior caval venous blood to the right heart, pose a particularly unfortunate combination of small, poorly perfused lungs, impaired cardiac filling and hypoplasia of left heart structures [3]. This combination may not benefit much from FETO, as the high intrathoracic pressure and the lack of available intrathoracic space do not allow for sufficient lung distension by this approach which may further impair cardiac filling. Therefore, we attempt to develop alternative therapeutic strategies for this severely affected patient group.

Iatrogenic fetal laparoschisis was proposed many years ago as a possible treatment for severe diaphragmatic hernia and has even been performed by open fetal surgery in a desperate case [4,5]. It has successfully been tested by open and—more recently—by fetoscopic surgery in a fetal sheep-diaphragmatic hernia model [5,6]. Therefore, when we were once again confronted with a humen fetus with a grave prognosis, we decided to offer the fetoscopic approach as a last resort.

Based on our longstanding clinical experience with the management of complex fully percutaneous fetoscopic setups in humans [7,8], and previous own studies in fetal sheep [9], we combined FETO with fetoscopic fetal laparoschisis in order to partially eventerate fetal bowel. The purpose of our case report is to present the technique, outcome and implications of this novel therapeutic approach, which we term “FETO-LAP”.

### Case

**Figure 1:**
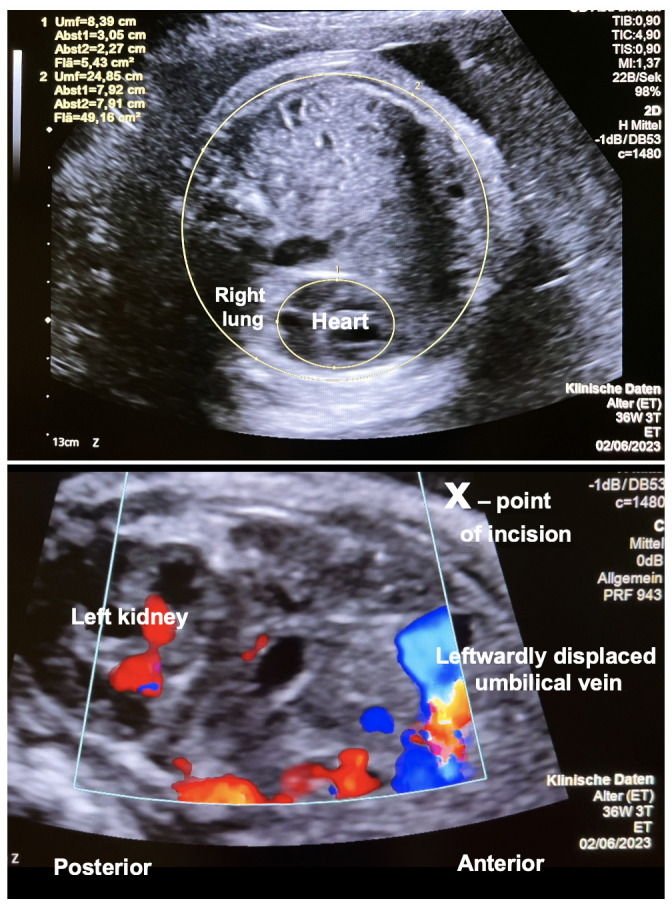
Cross-section through the chest in a human fetus with severe left-sided diaphragmatic hernia at 36 + 3 weeks + days of gestation. (**Top**)—Note the small area left for the fetal heart and right lung as a result of the large volume of herniated abdominal organs. (**Bottom**)—Oblique horizontal view of the upper fetal abdomen. This view was taken in order to follow the course of the intraabdominal umbilical vein. Note the close proximity of the left kidney, the leftwardly displaced coursing umbilical vein and the incision site (x). The left lateral vertical incision avoids the risk of injury to the larger abdominal wall vessels.

A 27-year-old pregnant woman (gravida 1, para 0) was admitted to our center at 36 + 3 weeks of gestation. In her singleton fetus, a severe left diaphragmatic hernia with an o/e LHR of ≤25% and an MRI lung volume of ≤20%, as well as poor pulmonary perfusion and marked left heart hypoplasia from preferential streaming of ductus venosus/inferior caval vein flow toward the right side of the heart, was observed (Figure 1—top).

After interdisciplinary counselling of both parents about the limited chances of neonatal survival without any prenatal treatment, the limitations expected from performing FETO as the only measure in such a case, and the potential benefits and maternal and fetal risks, as well as the experimental nature of combining FETO with the fetoscopic partial removal of the herniated bowel from the fetal chest by fetoscopic laparoschisis (FETO-LAP), the parents consented to the experimental intervention.

According to German law, as an individual, potentially life-saving treatment attempt, no approval by our local ethics committee was required. Apart from this deviation, the procedure was performed according to the guidelines of the Helsinki declaration. Importantly, there were no experimental surgical steps required for any maternal part of the intervention, as all necessary techniques had been routinely in use with low maternal and fetal risks for more than 15 years at our center.

**Figure 2:**
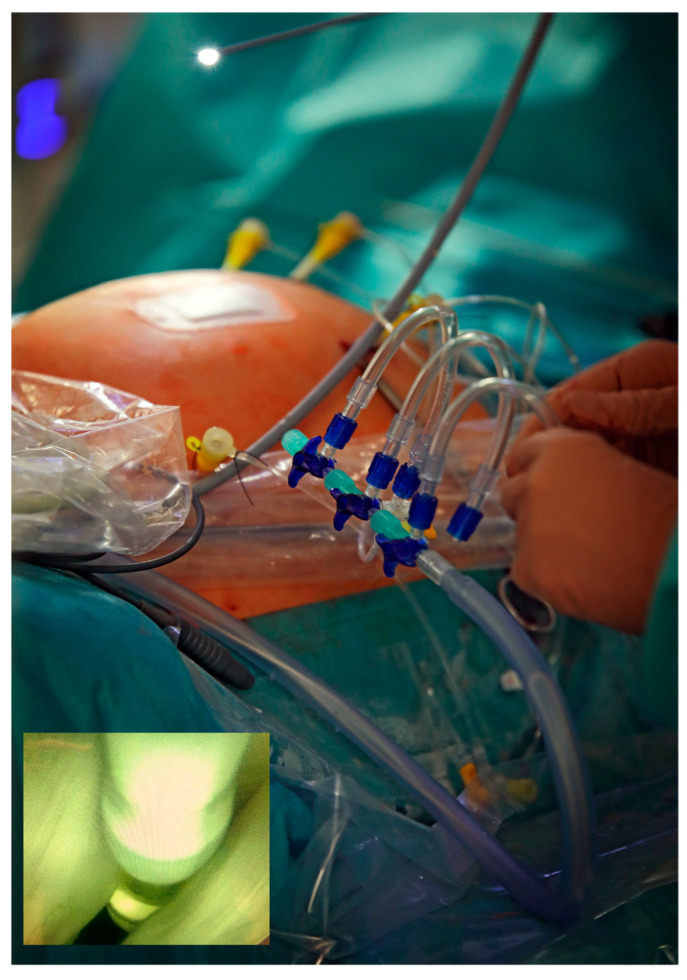
External aspect of the fully percutaneous, minimally invasive fetoscopic setup that required four short skin incisions and four percutaneous–transabdominal–transuterine–paraplacental punctures with 18-gauge needles (external diameter 1.2 mm) for over-the-wire insertion of four 11-F-canulas (external diameter 5 mm). Bottom left—Fetoscopic view during fetoscopic tracheal balloon occlusion, moments before detaching the latex balloon.

The procedure was performed under general maternofetal anesthesia at 36 + 5 weeks of gestation. Intraoperative tocolysis was performed by the continuous infusion of the oxytocin antagonist atosiban. The infusion was stopped on the first day after surgery. Adequate preparations for performing an emergency C-section at any time if needed had been taken. Following the intraamniotic ultrasound-guided insertion of an 11-F-sheath (external diameter 5 mm) close to the fetal mouth, a fetoscope was advanced into the fetal trachea. Via the working channel of the fetoscope, the fetal trachea was occluded with a commercially available latex balloon (Figure 2).

Guided by maternal transabdominal ultrasound imaging, three additional 11-F-sheaths were inserted into the amniotic cavity and percutaneous partial amniotic carbon dioxide insufflation (PACI) was carried out as previously described [3,7] (Figure 2).

After intraamniotic insertion of a 3.3 mm rod-lens fetoscope, the fetus was postured on its right side with endoscopic instruments.

**Figure 3:**
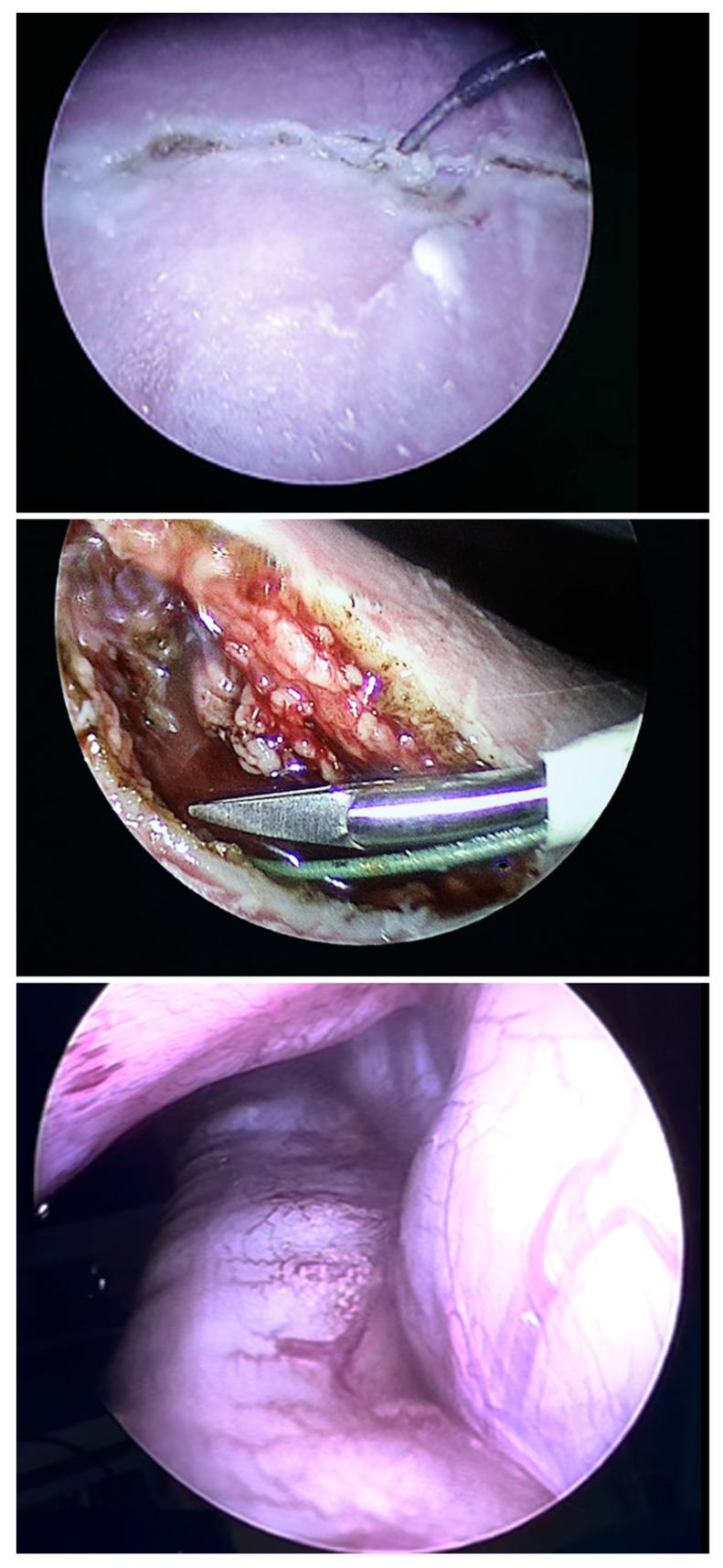
Employing monopolar cautery (**top**), scissors (**middle**) and various graspers, a vertical laparotomy was performed in the anterior axillary line, thus transsecting the oblique and transverse abdominal muscles. Through the opening, it was easily possible to introduce the fetoscope via the defect into the fetal chest and even insufflate the fetal thoracic cavity (**bottom**), gaining superior visibility of the herniated small and large bowel (**bottom**).

Employing scissors, monopolar cautery and various graspers, a vertical laparotomy was performed in the anterior axillary line, thus transsecting the oblique and transverse abdominal muscles (Figure 3). Particular care was taken to stay intraperitoneally and anterior to the left kidney. However, as a result of the leftward shift of the liver hilus from the intrathoracic herniation of its left lobe, the greatest care had to be taken not to injure the intraabdominal portion of the umbilical vein that coursed in immediate proximity below the incision site (Figure 1).

After 2 h of slow and careful dissection, interrupted by holding PACI and refilling the amniotic cavity for ultrasound assessment of the progress, orientation and depth of the incision in relation to the course of the intraabdominal umbilical vein, we succeeded in creating a 4 cm long vertical laparotomy in the anterior axillary line. Through the opening, it was easily possible to introduce the fetoscope via the defect into the fetal chest and even briefly insufflate the fetal thoracic cavity (Figure 3).

**Figure 4:**
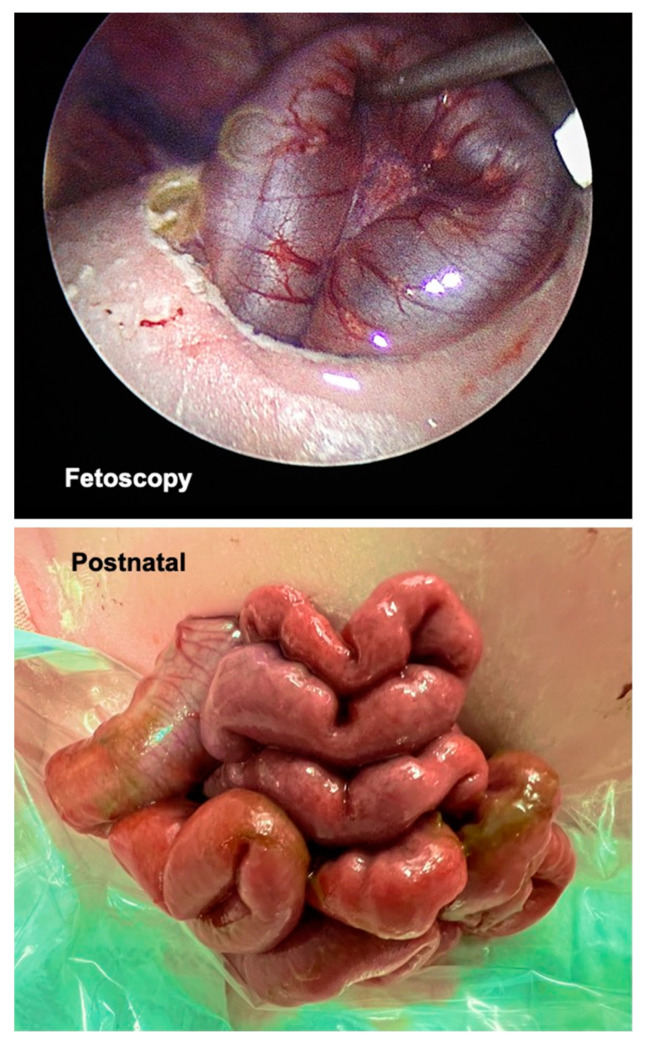
Fetoscopic view during FETO-LAP at the moment of eventeration of one bowel loop before the fetal abdomen (**top**). Neonatal aspect of the herniated bowel loops in the delivery suite (**bottom**).

After the successful eventeration of one bowel loop before the fetal abdomen (Figure 4—top), the insufflation was halted and the amniotic cavity refilled with warmed, sterile crystalline solution. Following trocar removal, the maternal abdominal skin incisions were closed with single stiches.

Mother and fetus tolerated the entire procedure well; no complications were observed during surgery. The pregnancy could be upheld for four more days when spontaneous contractions occurred at 37 + 2 weeks of gestation, and the child was delivered by caesarean section. Still on placental support, the tracheal balloon was removed and the baby was stabilized in the delivery suite and placed on ECMO in the first hours of life.

In the four days between FETO-LAP and delivery, more bowel loops had herniated through the iatrogenic abdominal wall defect (Figure 4—bottom). The exposed bowel of the neonate was placed within a sterile plastic bag. Over the next eight days, the patient could be managed without systemic infection or signs of bowel ischemia. On lung support, a rapid increase in tidal volume was seen over the next eight days. Unfortunately, after this period, blood clots obstructed the ECMO circuit and the neonate passed away.

## 2. Discussion

Despite the widespread use of FETO in Europe, the postnatal survival rate of fetuses with severe diaphragmatic hernia is still poor [2,10]. Therefore, finding new routes and strategies for their prenatal treatment is important. Our case shows that in this condition, by using a fully percutaneous, minimally invasive fetoscopic approach, it is technically possible to remove the herniated bowel from the fetal chest cavity and transfer it into the amniotic cavity via a surgically created fetal laparoschisis.

Similar to previous experiences with a laparoschisis model in sheep [6,9], laparotomy and the eventeration of a small bowel loop may be sufficient for more loops of bowel to herniate through the abdominal wall defect into the amniotic cavity in the days after surgery. Importantly, the case also shows that the eventerated bowel can be managed well after delivery, and bleeding from the abdominal incision site is not an issue despite the use of anticoagulant medication during ECMO.

The strategy of clearing the herniated organs from the chest is derived from the work of the pioneer of fetal surgery, Professor Michael Harrison, who was the first to achieve this feat during complete diaphragmatic hernia repairs in human fetuses by open fetal surgery [11]. Unfortunately, in liver-up fetuses, the total removal of all herniated organs from the chest, followed by fetal abdominal closure, resulted in their demise. Kinking of the intraabdominal fetal umbilical vein was suggested as the most likely culprit [12]. In contrast, our case shows that the removal of the bowel alone from the chest cavity of a human fetus with a liver-up diaphragmatic hernia–for the first time by a minimally invasive fetoscopic approach–can be well tolerated and does not necessarily jeopardize its fetoplacental circulation.

### Implications

We performed the first case of FETO-LAP at a time when the commercial availability of current balloons is in jeopardy and new devices are not yet on the market. Given the rigidity of the European Medical Device Regulation, which results in massive additional costs for invention, production, certification and repeated recertification cycles, it remains to be seen whether new devices can be afforded. This situation makes the search for alternative treatment strategies even more urgent.

The minimally invasive fetoscopic approach can be performed with generally low maternal risks and good outcomes [7]. However, as FETO-LAP opens the door to a more invasive approach to prenatal surgery on fetuses with severe diaphragmatic hernias, once again, answers must be sought to the following questions:

- How high will the technical success rate be in a larger series?
- Which intraoperative fetal complications are possible?
- What is the risk of and what could be the reasons for fetal demise during or early after surgery?
- If the procedure was performed within a few weeks prior to delivery, would it facilitate neonatal resuscitation, lower the need for ECMO or shorten the time on ECMO by enabling one to obtain sufficient tidal volumes and alleviating pulmonary hypertension more quickly?
- Would it make sense to assess a staged approach around 30 weeks of gestation, with laparoschisis first and, if the effect is not sufficient, followed by FETO?
- If the procedure was performed around 24 weeks of gestation, would it achieve the better catch-up growth of the lungs and even obviate the need for FETO?
- Will any positive effects on the lungs that could be achieved after early treatment be offset by very early delivery without the availability of ECMO?
- If the procedure was performed early, would it have any detrimental effect on the development of the chest wall?
- Would the risk to the eventerated bowel be similarly low as in gastroschisis or would there be a high risk of severe bowel complications?
- What is the impact on postnatal management?
- To what degree will the new approach be met by resistance within the healthcare community?
- In the end, will such a strategy, once refined and developed, result in better postnatal survival, better neurological outcomes, lower rates of pulmonary hypertension, and improved quality of life for patients with severe or moderate diaphragmatic hernias?

There were a number of reasons for us to perform the first case near term: in our experience, favorable survival results can be achieved in fetuses with severe diaphragmatic hernia by using a deliberately delayed and shortened FETO approach [13]. Earlier, FETO alone did not seem a beneficial option because there was barely any space left within the chest for lung growth. Cardiac filling was already impaired. Any further distension of the intrathoracic volume by lung distension and growth by FETO might have further impaired fetal cardiac filling, with the potential risk of further impairing the fetal cardiac output.

Furthermore, at our center, higher survival rates after ECMO are seen when affected fetuses are delivered near term [14]. Lastly, we wished to perform the laparotomy on a more mature fetus, decreasing the risk of inadvertent injury to the abdominal wall vessels or the bowel during the learning phase of the procedure.

## 3. Conclusions and Lessons Learned

Our case shows that fetoscopic fetal laparoschisis (FETO-LAP) was possible in a near term human fetus with severe diaphragmatic hernia. Via the abdominal effect, a large portion of the bowel eventerated through the abdominal wall defect into the amniotic cavity. This strategy of clearing the herniated organs from the fetal chest may aid in improving the conditions for the catch-up growth of hypoplastic lungs. The approach was well tolerated by mother and fetus and did not impair the fetoplacental circulation. Particular attention must be paid to the close spatial relationship between the incision site, intraabdominal umbilical vein and left kidney. The new approach opens the door to a myriad of new therapeutic and basic research implications.

## Data Availability

Inquries regarding technical details of the procedure can be made to the corresponding author.

## References

[1] Ruano R., Yoshisaki C.T., Da Silva M.M., Ceccon M.E.J., Grasi M.S., Tannuri U., Zugaib M. (2012). A randomized controlled trial of fetal endoscopic tracheal occlusion versus postnatal management of severe isolated congenital diaphragmatic hernia. Ultrasound Obstet. Gynecol..

[2] Deprest J.A., Nicolaides K.H., Benachi A., Gratacos E., Ryan G., Persico N., Sago H., Johnson A., Wielgoś M., Berg C. (2021). Randomized Trial of Fetal Surgery for Severe Left Diaphragmatic Hernia. N. Engl. J. Med..

[3] Kohl T. (2023). Lifesaving Treatments for the Tiniest Patients—A Narrative Description of Old and New Minimally Invasive Approaches in the Arena of Fetal Surgery. Children.

[4] Porreco R.P., Chang J.H.T., Quissel B.J., Morgan M.A. (1994). Palliative fetal surgery for diaphragmatic hernia. Am. J. Obstet. Gynecol..

[5] Lynn D., Montgomery L.D., Belfort M.A., Saade G.R., Baker W., Pokorny W., Minifee P., Langston C., Jevon G., Van den Veyver I. (1995). Iatrogenic gastroschisis decreases pulmonary hypoplasia in an ovine congenital diaphragmatic hernia modell. Fetal Diagn. Ther..

[6] Bergholz R., Fromm F., Meinzer A., Muehlfeld C., Boettcher M., Wenke K., Tiemann B., Reinshagen K., Krebs T. (2021). Stereological Lung Parameters After Fetoscopic Abdominal Decompression of Congenital Diaphragmatic Hernia in an Ovine Model: A Pilot Study. J. Laparoendosc. Adv. Surg. Tech. A.

[7] Kohl T. (2014). Percutaneous minimally invasive fetoscopic surgery for spina bifida aperta. Part I: Surgical technique and perioperative outcome. Ultrasound Obstet. Gynecol..

[8] Schneck E., Koch C., Arens C., Schürg R., Zajonz T., Kohl T., Weigand M.A., Sander M. (2017). Anesthesiological Management of Fetal Surgery. Anasthesiol Intensiv. Notfallmed Schmerzther.

[9] Kohl T., Tchatcheva K., Stressig R., Gembruch U., Kahl P. (2009). Is there a therapeutic role for fetoscopic surgery in the prenatal treatment of gastroschisis?—A feasibility study in sheep. Surg. Endosc..

[10] Snoek K.G., Greenough A., Van Rosmalen J., Capolupo I., Schaible T., Ali K., Wijnen R.M., Tibboel D. (2018). Congenital Diaphragmatic Hernia: 10-Year Evaluation of Survival, Extracorporeal Membrane Oxygenation, and Foetoscopic Endotracheal Occlusion in Four High-Volume Centres. Neonatology.

[11] Harrison M.R., Adzick N.S., Longaker M.T., Goldberg J.D., Rosen M.A., Filly R.A., Evans M.I., Golbus M.S. (1990). Successful repair in utero of a fetal diaphragmatic hernia after removal of herniated viscera from the left thorax. N. Engl. J. Med..

[12] Harrison M.R. (1990). Fetal surgery. West. J. Med..

[13] Kohl T., Gembruch U., Filsinger B., Hering H., Bruhn J., Tchatcheva K., Aryee S., Franz A., Heep A., Müller A. (2006). Encouraging early clinical experience with deliberately delayed temporary fetoscopic tracheal occlusion for the prenatal treatment of life-threatening right and left congenital diaphragmatic hernias. Fetal Diagn. Ther..

[14] Stevens T.P., Chess P.R., McConnochie K.M., Sinkin R.A., Guillet R., Maniscalco W.M., Fisher S.G. (2002). Survival in early- and late-term infants with congenital diaphragmatic hernia treated with extracorporeal membrane oxygenation. Pediatrics.

